# Role of hydroxylation for the atomic structure of a non-polar vicinal zinc oxide

**DOI:** 10.1038/s42004-020-00442-6

**Published:** 2021-01-20

**Authors:** Elin Grånäs, Michael Busch, Björn Arndt, Marcus Creutzburg, Guilherme Dalla Lana Semione, Johan Gustafson, Andreas Schaefer, Vedran Vonk, Henrik Grönbeck, Andreas Stierle

**Affiliations:** 1 Deutsches Elektronen-Synchrotron (DESY), 22607 Hamburg, Germany; 2 Department of Physics and Competence Centre for Catalysis, Chalmers University of Technology, 412 96 Göteborg, Sweden; 3 Fachbereich Physik, Universität Hamburg, 20355 Hamburg, Germany; 4 Division of Synchrotron Radiation Research, Lund University, 221 00 Lund, Sweden; 5 Department of Chemistry and Chemical Engineering and Competence Centre for Catalysis, Chalmers University of Technology, 412 96 Göteborg, Sweden

**Keywords:** Materials for energy and catalysis, Surfaces, interfaces and thin films

## Abstract

From the catalytic, semiconducting, and optical properties of zinc oxide (ZnO) numerous potential applications emerge. For the physical and chemical properties of the surface, under-coordinated atoms often play an important role, necessitating systematic studies of their influence. Here we study the vicinal ZnO($10\bar{1}4$) surface, rich in under-coordinated sites, using a combination of several experimental techniques and density functional theory calculations. We determine the atomic-scale structure and find the surface to be a stable, long-range ordered, non-polar facet of ZnO, with a high step-density and uniform termination. Contrary to an earlier suggested nano-faceting model, a bulk termination fits much better to our experimental observations. The surface is further stabilized by dissociatively adsorbed H_2_O on adjacent under-coordinated O- and Zn-atoms. The stabilized surface remains highly active for water dissociation through the remaining under-coordinated Zn-sites. Such a vicinal oxide surface is a prerequisite for future adsorption studies with atomically controlled local step and terrace geometry.

## Abstract (web summary)

Under-coordinated corner and edge sites often dictate oxide properties, however, information about their atomic-scale structure and chemical nature is often missing. Here, a combined experimental and theoretical study of the ZnO(10-14) surface reveals stable, long-range ordered, non-polar facets of ZnO, with a high step-density and uniform termination.

## Introduction

Zinc oxide (ZnO) receives much attention owing to its exceptional catalytic, semiconducting, and optical properties^1,2^. In applications involving ZnO surfaces, under-coordinated sites like steps and edges play an important role^3^. To systematically study the influence of atomic step sites on the adsorption behavior as a function of the step-density or orientation, vicinal surfaces have proven very useful^4^. Such surfaces are composed of terraces of a low-index orientation separated by parallel steps in dense, highly ordered arrays. For metals there are numerous studies applying vicinal surfaces to elucidate the role of step edges in catalysis^4–7^. Vicinal oxides are, however, generally much less studied and are often poorly understood on an atomic-scale. Exceptions are Al_2_O_3_^8–12^ and SrTiO_3_^13–17^ for which several vicinal surfaces and their use as support for low-dimensional nanostructures was explored. Another example is vicinal TiO_2_, which has also been studied in some detail, providing insights in the stability and structure of steps and their influence on the electronic structure of the surface and in the role of step sites on the interaction with O_2_^18,19^. Despite the observation that the presence of surface steps on ZnO influences the decomposition and reaction products in heterogeneous catalysis^20–23^, a systematic investigation of vicinal ZnO surfaces correlating the structure and reactivity is lacking.

Owing to alternating layers of Zn^2+^ and O^2−^ in the nominal bulk-terminated ZnO wurtzite structure, many of the surface orientations are polar and thus unstable. The stabilization mechanisms for the polar, low-index ZnO (0001) and ($000\bar{1}$) surfaces were studied in detail, showing that the non-zero dipole moment of the surface is canceled by compensating the charge through reconstructions, leading to changes in the surface stoichiometry or by adsorbates, especially OH^24–37^. It was also proposed that hydrogen in subsurface sites contributes to the stabilization of the surface^38,39^. Hydroxylation at oxide surfaces is a generally observed phenomenon, for polar and non-polar surfaces alike. This raises the question which role hydroxylation has for ZnO. Understanding the effect of hydroxylation is also important for a better understanding of the shape and reactivity of ZnO nanoparticles. The most stable surface for Zn-terminated ZnO was suggested to be the ($10\bar{1}4$) surface^40–42^, a surface with an angle of 24.8^∘^ to the (0001)-surface. For the O-terminated ZnO surface the ($10\bar{1}\bar{4}$), with the same miscut angle as ZnO($10\bar{1}4$) but to the (000$\bar{1}$)-surface instead of (0001), is suggested as the most stable^43^. The bulk-terminated ZnO($10\bar{1}4$) is an oxide surface of Tasker type 2^44^, thus having zero total dipole moment of the surface unit cell. This high step-density vicinal surface thus is potentially stable owing to its lack of macroscopic dipole moment.

Here, we have employed a combination of surface X-ray diffraction (SXRD), scanning tunneling microscopy (STM), X-ray photoelectron spectroscopy (XPS), and density functional theory (DFT) to study the ZnO($10\bar{1}4$) surface. We find that this is a very stable ZnO surface with a potentially high reactivity due to the high density of under-coordinated sites. We identify the ZnO($10\bar{1}4$) surface to be bulk truncated, contrary to the earlier suggested nano-faceting model. Furthermore, our calculations show that OH groups are present on the surface even under the best ultra-high vacuum conditions at room temperature and additionally stabilize the ZnO($10\bar{1}4$) surface. This prediction is supported by a comparison of experimental O 1s core levels and theoretically calculated core level shifts for the clean ZnO($10\bar{1}4$) surface and in the presence of OH groups.

## Results and discussion

### Surface characterization with STM and low-energy electron diffraction (LEED)

**Fig. 1:**
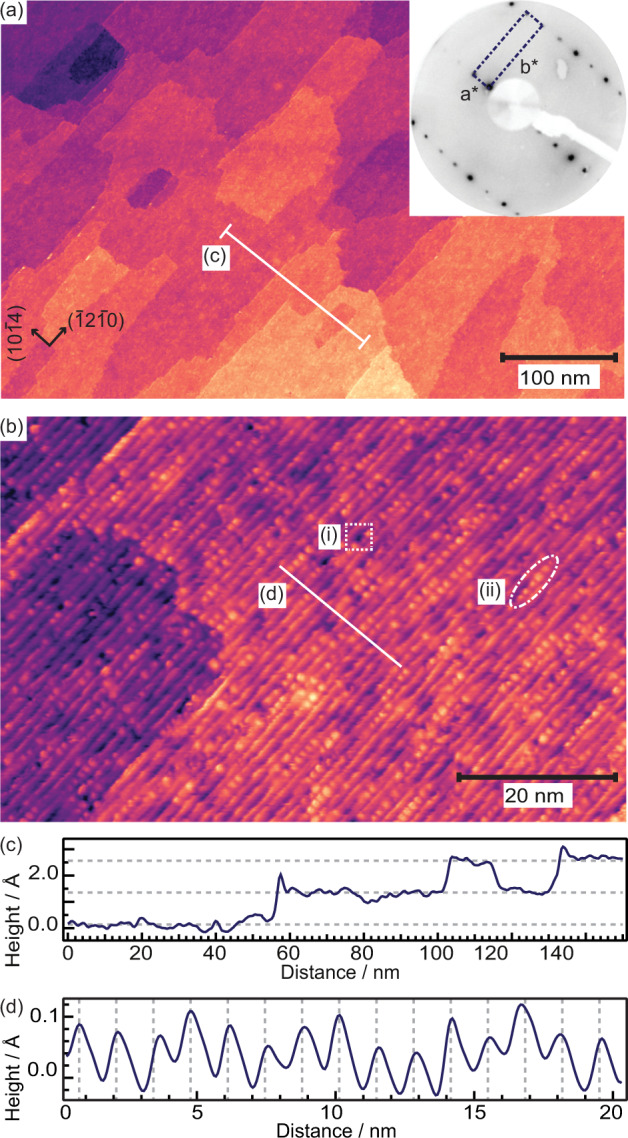
STM characterization of the ZnO($10\bar{1}4$) surface. **a** STM image of surface prepared by annealing for 10 min at 650 ^∘^C. 550 × 350 nm^2^, *I* = 0.2 nA, *U* = 1.8 V. Inset: LEED pattern of the ZnO($10\bar{1}4$)-surface (52 eV) with the surface unit cell marked by the dashed rectangle. **b** STM image of the as prepared ZnO($10\bar{1}4$) surface. 80 × 50 nm^2^, *I* = 0.2 nA, *U* = 1.8 V. Dotted squares: type (i) defects, dash-dotted ovals: type (ii) defects. **c** Line scan across several terraces along the line indicated (**a**). Averaged over 20 nm. Dashed lines indicate the determined average height between terraces: 1.2 Å. **d** Line scan perpendicular to the steps along the line indicated in **b**. Dashed lines indicate the average step periodicity of 13.1 Å.

Figure 1(a) shows a representative STM image of the as prepared ZnO($10\bar{1}4$) surface. The surface consists of large terraces (avg. size 1080 nm^2^) elongated in the step edge direction. The average surface roughness is 1.4 Å. As comparison, this is in between the previously observed roughness of <1 Å for the (0001), ($000\bar{1}$), and ($10\bar{1}0$), and 2.5 Å for the ($11\bar{2}0$) surface^24^. The steps of the ZnO($10\bar{1}4$) surface appear as stripes, better seen in the smaller STM image in panel (b). Analyzing a large number of images from different preparations we find the height between terraces to be 1.2 ± 0.1 Å and the step periodicity 13.1 ± 2.4 Å. The terrace height can be seen in the line scan in panel (c) [taken along the line in panel (a)]. The dashed lines indicate the determined height between terraces. The separation between the terraces is identical to the theoretical value of the distance between lattice planes for the ZnO($10\bar{1}4$) surface, 1.18 Å, in line with a uniform surface termination. In Fig. 1(d) a line scan taken perpendicular to the steps in panel (b) show the step periodicity. The step periodicity is in good agreement with the theoretical value *a* = 12.4 Å (*b* = 3.29 Å) and with that previously observed by Zheng et al.^40^ on the faceted ZnO(0001) surface. For vicinal surfaces with periodic arrays of steps the LEED pattern is expected to exhibit a spot splitting around the fundamental signals of the low-index surface, in this case (0001), where the separation between the spots are the reciprocal of the average periodicity of the vicinal surface^45^. The inset in panel (a) shows the LEED pattern of the as prepared ZnO($10\bar{1}4$) surface, where the spot-slitting expected for a non-reconstructed ZnO($10\bar{1}4$) surface can clearly be seen. The well-defined spots and the absence of smearing indicate that the surface is both well-ordered on a larger scale and has a narrow distribution of step widths. We have discussed the LEED pattern of the ZnO($10\bar{1}4$) surface in more detail in a previous publication.^42^

**Fig. 2:**
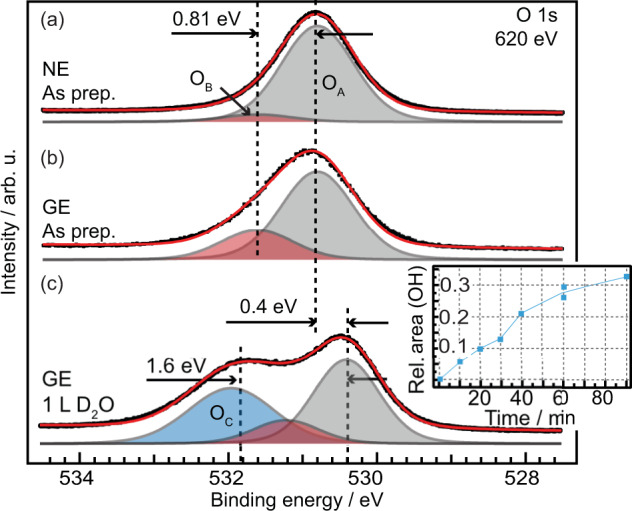
Measured O 1s core level spectra. **a** O 1s of ZnO($10\bar{1}4$) for the as prepared surface measured in normal- and **b** grazing emission. **c** O 1s after exposure to 1 L D_2_O at room temperature, grazing emission. Black dots: measured data. Red line: fitted spectra. Shaded peaks: fitted components as labeled in the figure. Inset: Peak area of the XPS O 1s O_*C*_ component, normalized to the water saturated spectrum, as a function of time in 1 ⋅ 10^−10^ mbar residual gas, mainly composed of H_2_ and H_2_O. Line to guide the eye.

Two types of defects are also seen in Fig. 1(a): (i) dark points appearing as “dents” on the steps and (ii) bright protrusions along the steps. An example of type (i) is marked by a dotted square and type (ii) by a dash-dotted oval. Though the lack of atomic resolution makes it hard to draw any definite conclusions we attempt to assign the defects as follows. The concentration of type (i) defects does not change with time in vacuum, after exposure to O_2_ (see Fig. S1 and surrounding discussion), or after repeated scanning in the same area. Based on their inertness we tentatively assign these defects to Zn- or ZnO vacancies. This is in line with studies on the ($10\bar{1}0$) ZnO surface, where ZnO vacancies have been shown to be the thermodynamically most favorable type of atomic defect^46^. Type (ii) defects, in contrast, slowly increase in number with time when the sample is kept at room temperature, indicating that they are due to adsorbates. We assign them to OH accumulating on the surface during the measurement times (up to 10 h), despite very good vacuum conditions in the low 10^−11^ mbar regime. Adsorption of OH from the background pressure is in line with our XPS measurements (see Figs. 2 and S2) and DFT calculations, which indicate that partially dissociative water adsorption at the ZnO($10\bar{1}4$) surface is strongly exothermic. Similar to our STM observations on the ZnO($10\bar{1}4$) surface, two types of defects were observed with atomic force microscopy and assigned to Zn-vacancies and H adsorbed on Zn-atoms, respectively, on a reconstructed Zn-terminated ZnO(0001) surface^33^.

**Fig. 3:**
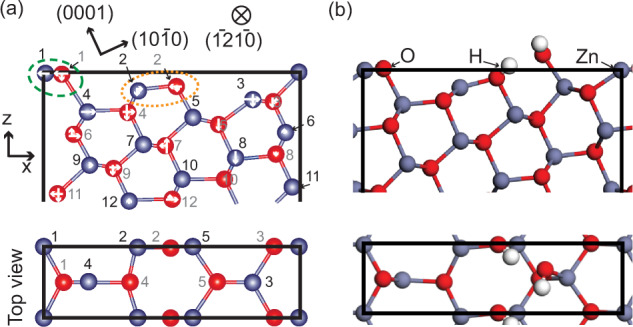
Structural models of the ZnO($10\bar{1}4$) unit cell. **a** The bulk truncated surface model. White arrows indicate the fitted displacements. Atomic colorcode: H (white), O (red), Zn (blue). Orange dotted oval: Zn-O pair removed for Model B, Green dashed oval: Zn-O pair removed for Model C. Numbers correspond to the labels in Table SI. **b** DFT optimized structure based on Model A, including one dissociated H_2_O molecule per unit cell.

Based purely on our STM measurements we cannot exclude that some of the defects are subsurface hydrogen, as has previously been suggested to occur on ZnO^38,39,47^ and also observed on hydroxylated TiO_2_^48^. However, significant amounts of subsurface OH can only form from atomic hydrogen^47,49^ and in our case the only source of hydrogen is from dissociated H_2_O and H_2_ from the residual gases. In a study by Hellström et al^38^. subsurface OH was found on a surface not previously exposed to atomic hydrogen; in this study they measured a significant XPS component corresponding to OH core level shifts (CLS) of +1.5–2 eV with respect to the oxide component on the freshly cleaned surface. This was attributed to OH in the second layer, which contribute to the stabilization of the $c(\sqrt{12}\times \sqrt{12})$ R30^∘^ surface reconstruction. The OH component observed by Hellström et al. corresponds to our O_*C*_ component, and as we do not observe the O_*C*_ component on the freshly cleaned surface (see Figs. 2 and S3 and discussion thereof) we exclude significant amounts of subsurface OH in the near surface region in our case. Subsurface OH was also suggested in a X-ray standing wave (XSW) study of the hydroxylated ZnO(0001) surface^39^. Here, they observe a large in-plane disorder of the oxygen and suggest that OH has substituted oxygen lattice sites in the near surface region, as well as populate sites along the edges of triangular islands on the surface. Our results may be in line with this finding despite our exclusion of subsurface OH. In our final model [see Fig. 3(b)] we observe two OH species with different in-plane coordinates, such species would appear as in-plane disorder in XSW measurements.

### Investigation of the surface chemical composition by XPS

To obtain chemical information of the surface also XPS measurements were performed. The O 1s spectra of as-grown ZnO($10\bar{1}4$) are shown in Fig. 2 measured in (a) normal emission [NE] and (b) grazing emission at 60^∘^ [GE]. The O 1s XP-spectra for both measurement geometries can be de-convoluted in two components of identical position and width: the main component, O_*A*_ at 530.8 eV assigned to O^2−^ in wurtzite ZnO^30,50–52^, and a second component, O_*B*_, shifted +0.81 ± 0.03 eV from the O_*A*_ component. The shift of O_*B*_ is significantly lower than the 1.4–2.1 eV that was reported for OH adsorbed on low-index ZnO surfaces^30,50,52,53^. The relative intensity of the O_*B*_ component is increasing when going from NE to GE (from 7 % to 33 %), indicating that it is attributed to surface oxygen species.

The surface was also exposed to 1 L D_2_O at room temperature (3.5 ⋅ 10^−10^ mbar). The spectrum after exposure is shown in Fig. 2(c), where a large peak shifted +1.6 eV from the main peak is seen to appear. This new component, O_*C*_, we assign to OH and H_2_O adsorbed on the surface. We exclude that this peak corresponds to CO or CO_2_, see Fig. S3 for the corresponding C 1s spectra and further discussion. The O_*C*_ component is also seen to increase when a freshly prepared surface is exposured to ultra-high vacuum (UHV) for longer times, see inset in Fig. 2(c), through adsorption of water from the background pressure and we correlate this with the type (ii) defects seen in STM (see previous section). The as-prepared surface does not exhibit any O_*C*_ component, which would be expected if significant amounts of subsurface OH was present. The XP-spectra corresponding to the data points in the inset are shown in Fig. S2. After exposure to D_2_O a peak shift of 0.4 eV is observed for the O_*A*_ and O_*B*_ components in GE. In NE, however (see Fig. S4), the shift is 0.17 eV. All spectra are calibrated to the Fermi-level of a Cu(111) crystal in electrical contact with the ZnO($10\bar{1}4$) crystal. The Cu Fermi-level can be assumed to not be affected at these experimental conditions, and it can thus be excluded that the shift is an error in binding energy calibration. Instead we assign this peak shift to an adsorption induced metallization in the surface region, with a downward bending of the bands close to the surface. This is exclusively a surface phenomenon and only extends a few nanometers into the bulk^54^, which explains why the observed shift scales with the probing depth (the probing depth for the O 1s spectra in NE and GE is estimated to be 8.1 Å and 4 Å, respectively). Though the surface metallization is more pronounced for H-adsorption it was also observed for ZnO exposed to water and methanol^55^.

### Determination of surface structure using SXRD

To obtain information on the atomic structure of the ZnO($10\bar{1}4$) surface, surface X-ray diffraction experiments were performed. A set of crystal truncation rod (CTR) data was obtained from the as prepared surface, no signal from potential super-structures was detected. Three different models were tested against the CTR data and used as starting points for further refinement: A—the bulk truncated surface model seen in Fig. 3(a), B—the model suggested by Zheng et al.^40^ where one under-coordinated Zn-O pair is removed from the surface (indicated by the orange dotted oval), C—a model where the topmost under-coordinated Zn-O pair is removed from the surface (green dashed oval). The DFT relaxed structures of all three models are shown in Fig. S5. All three surfaces exhibit a mixed-terminated stepped structure with (0001)-planes at an angle of 24.8^∘^ to the surface and a periodicity of 12.4 Å perpendicular to the steps. In principle, the three models cannot be differentiated based on coordination; all these surfaces have the same amount of under-coordinated atoms. The positions of Zn- and O- atoms in the first seven layers along the surface normal were refined. Owing to the mirror symmetry of the structure, the atoms’ y positions were kept fixed and the x- and z-positions were allowed to vary. These directions are perpendicular to the steps and along the surface normal, respectively, as indicated in Fig. 3(a). The weighted squared difference between the measured and calculated structure factors, *χ*^2^, was minimized and its value used for comparison between the different models. The best fits were obtained by accounting for an atomic-scale r.m.s. roughness of 1.4 Å^56^, in very good agreement with the STM measurements.

After optimization of the atomic displacements in the x- and z-directions the agreement between measured and calculated structure factors, *χ*^2^, are 1.65, 2.95, and 2.14 for Models A, B, and C, respectively. The final fits were obtained by fixing the z-position of O-atoms 2 and 14 and the x-position of O-atoms 2, 5, and 9 to their bulk positions because it was found that the data are not particularly sensitive to these parameters, resulting in nonphysical bond lengths. The ranking of the models, with Model A corresponding best to the experimental surface structure and Model B the worst, is consistent with the ranking prior to fitting (see the first two columns in Table 1) and was found to hold for all the different approaches we tried. Thus, even with relatively large errors on the oxygen atomic positions the distinction between the three surface models, that differ in particularly in their surface composition with one Zn-O pair being missing or present, is robust. The full set of CTR data obtained from the as prepared surface is shown in Fig. S6 together with the calculated structure factors for the three models. The atomic displacements for the best fit of Model A are shown in Table S1. The most significant displacements in both the x- and z- directions are observed in the first four layers where the atoms are either under-coordinated or directly neighboring under-coordinated atoms.

**Table 1:** Comparison of the three models fitted against the SXRD data and used as a starting point in the DFT calculations.

	*χ*^2^				Δ*σ* [meV/Å^2^]		
	Unrelax.	Bare	Occup.	Addit. O	Bare	H_2_	H_2_O
Model A	5.04	1.65	1.63	1.52	0	−29	−48
Model B	9.76	2.95	–	3.23	12	−30	−47
Model C	5.59	2.14	–	2.17	1	−13	−34

The normalized goodness-of-fit, *χ*^2^, from the SXRD fits are given for four different cases: (Unrelax.) the unrelaxed models of the adsorbate-free surface, (bare) the adsorbate-free, bare surface after relaxation, (Occup.) with the occupancy fitted for the two top Zn-O groups as explained in the main text, and (Addit. O) for one additional O-atom adsorbed on the surface (hydrogen atoms are not visible by X-rays). The surface energies (Δ*σ*) from the DFT calculations are also given for three different situations: the bare surface, with dissociated H_2_, and with dissociated H_2_O.

Starting from the optimized Model A, also the occupancy of the Zn-O pairs corresponding to Model B and C, as indicated in Fig. 3(a), were fitted. The occupancy is fitted to be 0.98 and 1.00, respectively. An occupancy of 1 for both these Zn-O pairs correspond to the bulk-terminated Model A. Fitting the occupancy only gave minor improvements in the *χ*^2^, from 1.65 for the pure Model A to 1.62.

To summarize the SXRD results: Model A, having the lowest *χ*^2^ value, shows the best correspondence to the experimental surface structure. Models B and C can, therefore, already be ruled out on the grounds of the SXRD data analysis.

### DFT calculations and comparisons to experiments

To further analyze our assignment of the surface structure DFT calculations were performed. The surface energies at room temperature referenced to the bare surface (structural model A) surface are shown in Table 1. Model B was found to have a considerably higher surface energy than the other two models, which have similar stability. This is consistent with the SXRD fits (also shown in Table 1) that show that Model B has the worst agreement with the experimental data.

**Fig. 4:**
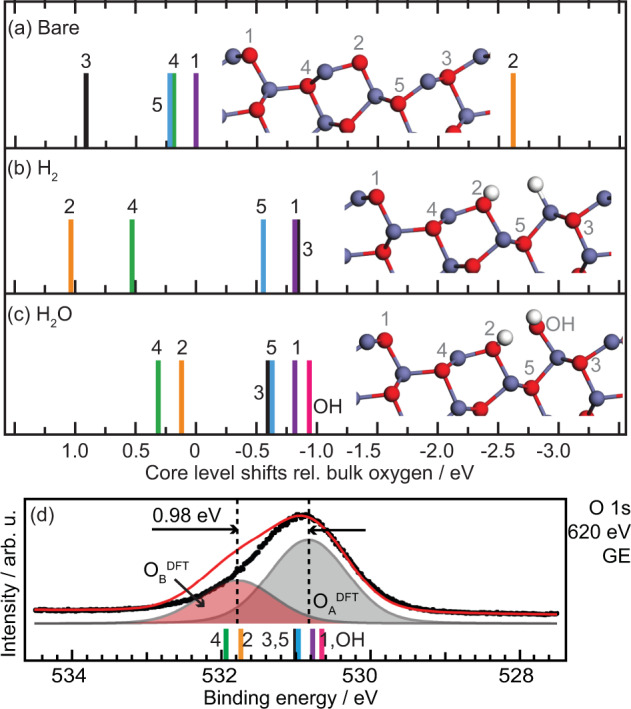
Calculated O 1s core level shifts of Model A. **a** Calculated O 1s core level shifts for Model A without adsorbates, **b** with H_2_-, and **c** H_2_O adsorbates. Numbers refer to atoms as indicated in the structural models. Atomic colorcode: H (white), O (red), Zn (blue). In both cases the coverage was one molecule per surface unit cell. **d** Simulated XP-spectra (red line) based on the CLS shown in panel **c** compared to the measured O 1s in GE (black dots). The measured data is the same as in Fig. 2(b).

To corroborate the assignment of the model for the surface structure, the O 1s CLS were calculated and compared to experiments. The CLS for Model A are shown in Fig. 4(a)–(c), whereas the results for Model B and C are given in Fig. S7. For all three bare surfaces we obtain a large negative CLS for O-surface atoms coordinated by only two Zn-ions for all three bare surface models. Such a negatively shifted component is not observed in the measured O 1s XP-spectra in Fig. 2(a) and (b).

Attempting to explain the absence of a peak at lower binding energies, we also performed calculations for adsorption of two common residual gases in vacuum, namely hydrogen and water. In both cases the coverage was one molecule per surface unit cell. The adsorption of both molecules has a significant influence on the position of the CLS, see Fig. 4(b) and (c). For the energetically more favorable dissociative water adsorption, the presence of OH-groups results in the shift of O 1s CLS of the oxygen adjacent to the adsorbate (position 3 in Fig. 4), from 0.9 to −0.6 eV versus bulk oxygen. This shift is consistent with the formal destabilization of the adjacent Zn ion by the presence of the additional OH-group. A comparable but smaller shift towards lower binding energies is also observed for the second O (position 5 in Fig. 4) adjacent to the destabilized Zn ion. The under-coordinated O, with a relative binding energy of −2.6 eV for the bare surface is through water adsorption converted into an OH group and shifted in the opposite direction to 0.1 eV versus ZnO bulk (position 2). Interestingly, an even more negative CLS of −0.9 eV is calculated for the second OH-group. The very negative relative CLS of the OH group coordinated to only one Zn-cation at the ZnO surface is different from what was observed earlier at more ionic oxides surface, such as MgO(100) where OH groups appear at a CLS of approx. +2 eV^57^ with respect to the bulk oxygen. The negative shift with respect to the bulk oxygen is a consequence of efficient screening of the core hole and stabilization by hydrogen-bonding. A discussion about the differences between ZnO and MgO can be found in the Supplementary Information, section “Comparison to O 1s in MgO(100)” (Fig. S8). The positive CLS calculated for position 2 and 4 relative to the other positions after adsorption of H_2_O indicate that a second O-component should be present in the XP-spectra, in line with the observed O_*B*_ component. Note that the O-atoms in position 2 and 4 are part of the ZnO-structure, not additional OH. The OH-group adsorbed on the surface after H_2_O dissociation has a CLS that place it close to the O-atom in position 1. For the less favorable H_2_ adsorption (adsorption energy of 1.12 eV) the CLS are similar to the H_2_O-case, however, with a larger spread that matches the experimental observations less well than H_2_O adsorption.

In Fig. 4(d) the spectrum simulated from the CLS for 1 H_2_O on Model A is compared to the measured spectrum. The simulated spectra were obtained by introducing six components, one for each oxygen atom, with identical intensity and width, and the relative CLS fixed to the calculated values. The width of the components was fitted, as was the BE position relative to the measurements. After fitting of the width and relative position the six components were summarized into two groups, as presented in Fig. 4(d): O${\,}_{A}^{{\mathrm{DFT}}}$ composed of oxygen in position 1, 3, 5, and OH, and O${\,}_{B}^{{\mathrm{DFT}}}$ composed of position 2 and 4. The calculated spectrum is in good agreement with the one measured, aside from the higher intensity and larger shift of the O_*B*_ component in the calculated spectrum. Both these differences can be attributed to that the XPS measurements, though extremely surface sensitive, include near surface atoms (probing depth ~ 4 Å in GE) that are not taken into account in the calculated spectra. Contributions from subsurface atoms may overlap with the calculated components, we can however not resolve this in the measurements. Note also that the high surface sensitivity of the XPS measurements means that we do not observe the bulk O1s reference species with "0” core level shift.

Thus, we have concluded from the SXRD measurements that Model A agrees best with the surface structure obtained experimentally. The DFT calculations confirm that this is a stable surface structure. However, comparing with the DFT calculations we see that the XP-spectra can only be explained by the presence of one dissociated H_2_O molecule per surface unit cell on one Zn- and O- site. The structure for molecular and dissociate water adsorption obtained here have similarities to the ones previously reported for defected ZnO(0001)^34,58^ and also to other oxide surfaces, in particular with regards to the OH group binding in a bridge configuration between two Zn-atoms and the H-ion occupying an oxygen site. Surprisingly, we do not observe this OH-species experimentally as the CLS overlaps with those of other O-sites on the surface and care needs to be taken when drawing conclusions on whether the surface is clean or not based purely on XPS measurements. The DFT calculations indicate that the adsorption and dissociation of water at the twofold-coordinated O (position 2) and the adjacent under-coordinated Zn ion is strongly exothermic and correspondingly results in a significant stabilization. The adsorption energies are strong on both Model A and B, making them energetically similar.

Revisiting the CTR data with the dissociated water in mind an O-atom corresponding to that of the OH-group was added to Models A and B as these model were found to be energetically similar in the DFT calculations. For both models the addition of one O-atom lead to some adjustments of the atomic displacements particularly in the first layer, however, the changes are comparatively small as the sensitivity to O-atoms in the SXRD measurements is low relative to the Zn-atoms. For Model A the additional O-atom and relaxation result in a slight improvement of the *χ*^2^ to 1.52 (from 1.65). For Model B and C on the other hand, the addition of an O-atom lead to a worse *χ*^2^. See also Table 1, column 4, and further discussion in the Supplementary Information, section “SXRD fit with additional O-atom” (Fig. S9 and Table S2).

**Fig. 5:**
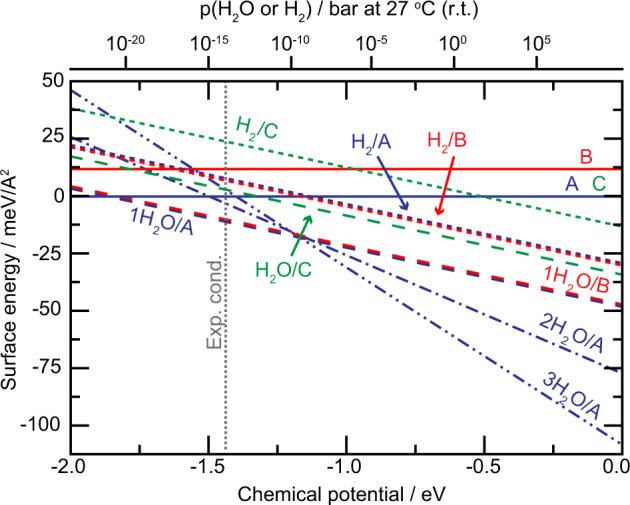
Phase diagram for the bare, water, and hydrogen covered surface models. The surface stability for bare, water and hydrogen covered A (blue), B (red), and C (green) surface models are displayed. All surface models are considered for the case of a bare surface (line), one adsorbed water molecule (dashed line) and one adsorbed hydrogen molecule (dotted line). Additionally, Model A is considered with two and three water molecules (dash-dotted line and dash-dot-dotted line, respectively). The energies are reported with respect to the bare surface energy of Model A. The chemical potential corresponding to the experimental conditions during surface analysis (−1.44 eV) is indicated by a vertical line.

To attempt to explain why the clean bulk-terminated surface is not stable even under the best UHV conditions at room temperature and further explore the presence of adsorbates on the surface a phase diagram was constructed (Fig. 5) using ab intio thermodynamics^59^. The relative surface energies shown in Table 1 are taken at the line indicating the experimental conditions. As mentioned above, the bare Model B shows considerably higher surface energy than the other two models, which exhibit similar stability. For all three surfaces dissociative adsorption of one water molecule is favored over one hydrogen molecule. Focusing on the energetically more favorable water adsorption we find that the adsorption energy of one water molecule is 1.85, 2.30, and 1.35 eV on Model A, B, and C, respectively. The high adsorption energy is related to the under-coordinated oxygen atom, yielding dissociative adsorption with a hydrogen bond between the two OH-groups, see Fig. 3(b). The calculated atomic displacements are shown in Table S3. For Model A, we also investigated the possibility with two and three water molecules and the adsorption energy per water molecule is calculated to be 1.50 and 1.40 eV, respectively (see Fig. S10 for structures). For Model A, the situation with one adsorbed (dissociated) molecule is preferred between water chemical potentials of −1.85 eV and −1.15 eV. At higher chemical potentials three molecules are preferred. We note that the thermodynamically stable phase during the experimental conditions is Model A with one adsorbed water molecule, in agreement with the previous conclusions.

Thus, we have shown that the ZnO($10\bar{1}4$) is a long-range ordered vicinal oxide surface. Using SXRD, XPS, STM, and DFT, we determine that the surface has a bulk truncated structure with OH present on two uncoordinated sites under all our experimental conditions. DFT shows that this dissociated H_2_O further stabilizes the surface significantly without altering the surface structure. The surface remains highly active for water adsorption and dissociation through the two remaining under-coordinated Zn-atoms also when it is partially covered by OH-groups. The experimentally observed core level shifts for the as prepared surface can consistently be explained by DFT. Such a surface with a high density of under-coordinated step sites is an ideal playground for future studies of how these sites influence the catalytic properties of ZnO.

## Methods

### Sample preparation

The ZnO($10\bar{1}4$) surface was cleaned through cycles of sputtering (500 eV Ar^+^, 20 min, room temperature (r.t.)) and subsequent annealing at 650^ ∘^C (residual pressure p_*R*_ < 3 ⋅ 10^−9^ mbar). To determine the optimal annealing temperature the terrace size was studied as a function of the annealing temperature and time by STM (see Supplementary Methods, Fig. S1(a)). The surface order and the concentration of defects was not seen to improve by annealing to temperatures over 650 ^∘^C or longer than 10 min. The terrace height is always ~1.2 Å. Exampe STM images taken after annealing to 630, 670 ^∘^C and after O_2_ annealing at 670 ^∘^C are shown in Fig. S1(b)–(d).

### Scanning tunneling microscopy (STM)

The STM measurements were performed in a Scienta Omicron VT SPM situated at the DESY NanoLab^60^. All measurements were performed at room temperature in constant current mode using etched W-tips (Ar-sputtered and annealed after introduction to UHV). The residual pressure in the chamber was p_*R*_ < 5 ⋅ 10^−11^ mbar. The temperature was measured by a thermocouple close to the sample, which was cross-calibrated at an earlier time with a thermocouple spot-welded to the side of a metal crystal mounted on the same type of plate as used for the ZnO sample. The images were post processed using Gwyddion ^61^.

### X-ray photoelectron spectroscopy (XPS)

XPS measurements were carried out at the former i311 beamline at the MAX IV Laboratory (p_*R*_ < 1 ⋅ 10^−10^ mbar)^62^. No contaminants were observed by XPS on the surface after cleaning (see Fig. S11). O 1s spectra were collected in both normal- (NE) and grazing emission (GE) at 60^∘^ from the normal with 620 eV photon energy with a total energy resolution of 130 meV. The O 1s XP-spectra were curve fitted using convoluted Doniach-Šunjić and Gaussian functions with a linear background and no asymmetry. During fitting the Lorentzian full width at half maximum for all components was fixed to 0.18 eV, in accordance with ref. ^63^. Further, the Gaussian full width at half maximum (GFWHM) of the O_*A*_ and O_*B*_ components were restricted to be the same. The GFWHM for O_*A*_ and O_*B*_ was found to be 1.1 eV, while O_*C*_ has a GFWHM of 1.3 eV. The binding energy position of the O_*B*_ component was determined through difference spectra between the normal and grazing measurements.

### Surface X-ray diffraction (SXRD)

The SXRD measurements were performed on the surface diffraction beamline ID03 at the European Synchrotron Radiation Facility (ESRF)^64^, using the dedicated UHV SXRD chamber (p_*R*_ < 1 ⋅ 10^−10^ mbar) and a photon energy of 18 keV. The SXRD data was analyzed using ROD from the ANAROD package^56^. During the structural refinement the atoms in the first seven layers along the surface normal were allowed to vary. Displacements in deeper layers have no or a very minor impact on the quality of the fit and were, therefore, kept fixed to their bulk values. During the fitting the Debye–Waller factors of all atoms were kept fixed to their bulk values of 0.651 and 0.64 Å^2^ for the Zn- resp. O-atoms^65,66^. Fitting or manually modifying the factors had no significant influence on the *χ*^2^ values. See Supplementary Information, section “SXRD models and fits” for further details.

### DFT calculations

Density functional theory (DFT) calculations were performed with the Vienna ab initio simulation package (VASP; version 5.4.1) using the PBE^67^ functional with a Hubbard U of 7.5 eV for the Zn 3d orbitals. The core electrons were treated within the projector augmented wavefunction (PAW) method^68^ and the Kohn–Sham orbitals were expanded in a plane wave basis, truncated at 550 eV. Properties of the ZnO($10\bar{1}4$) surface were obtained using a 13 monolayers thick slab where the bottom 9 monolayers were fixed to the bulk positions. slab of this thickness was required to obtain converged surface-bulk core level shifts. The calculations were done using a (1 × 1) surface unit cell together with a 1 × 5 × 1 k-point set. All structures were relaxed using the conjugate gradient algorithm implemented in VASP. The obtained lattice parameters of *a* = *b* = 3.19 Å and *c* = 5.12 Å are in good agreement with experiments^69^. Core level shifts were calculated with final state effects assuming complete screening of the core hole. In this method, the CLS is calculated as the total energy difference between the atom at consideration and a reference atom, which in our caseis a bulk atom. The core level shifts were calculated using a (1 × 3) surface cell with one k-point. The systems with a core hole were constructed by generating a PAW potential with an electron hole in the 1s shell of O. Charge neutrality was maintained in the calculations of the core level shifts by adding a jellium background^70^.

## Supplementary information

Supplementary Information

Description of Additional Supplementary Files

Supplementary Data 1

Supplementary Data 2

Supplementary Data 3

Supplementary Data 4

Supplementary Data 5

## Data Availability

Data not shown can be found in the Supplementary Information, the Supplementary Data files (Supplementary Data 1–4: structures used for the SXRD fits, Supplementary Data 5: measured and calculated structure factors for the best fitting model) or can be requested from the corresponding author.
