# Supplementary material for: Role of hydroxylation for the atomic structure of a non-polar vicinal zinc oxide
Source: Commun Chem. 2021 Jan 20;4:7. doi: 10.1038/s42004-020-00442-6 (PMC9814410; doi:10.1038/s42004-020-00442-6)
Supplement: Supplementary file 1 — Supplementary Information [file 42004_2020_442_MOESM1_ESM.pdf]

Supplementary information: Role of hydroxylation for the atomic structure of a non-polar vicinal zinc oxide

Elin Grånäs[1,+], Michael Busch[2], Björn Arndt[1,3], Marcus Creutzburg[1,3], Guilherme Dalla Lana Semione[1,3], Johan Gustafson[4], Andreas Schaefer[4,5], Vedran Vonk[1], Henrik Grönbeck[2], and Andreas Stierle[1,3,*]

[1]Deutsches Elektronen-Synchrotron (DESY), 22607 Hamburg, Germany
[2]Department of Physics and Competence Centre for Catalysis, Chalmers University of Technology, 41296 Göteborg, Sweden
[3]Fachbereich Physik, Universität Hamburg, 20355 Hamburg, Germany
[4]Division of Synchrotron Radiation Research, Lund University, 221 00 Lund, Sweden
[5]Department of Chemistry and Chemical Engineering and Competence Centre for Catalysis, Chalmers University of Technology, 41296 Göteborg, Sweden
[+]e.granas@protonmail.com
[*]andreas.stierle@desy.de

Supplementary Methods

STM study of the surface order

The surface order as observed by STM is sensitive to small temperature changes; for 10 min annealing times the average terrace size increased significantly with temperature between 580 °C and 650 °C, however no significant increase was observed for 670 °C as can be seen in Figure S1(a). Example STM images are shown in panel (b) and (c) for 630 and 670 °C. The pressure during annealing was in all cases better than $3 \cdot 10^{-9}$ mbar, however the pressure increased with annealing temperature and time. Neither doubling of the annealing time to 20 min, nor annealing for 10 min in $1 \cdot 10^{-7}$ mbar of O_2 at 580 °C or 670 °C affected the average terrace size. For the two higher annealing temperatures we also compared the concentration of type (i) defects (data not shown) and found no increase in defect concentration when the annealing temperature was increased from 650 °C to 670 °C. In both cases the concentration was 1-5 defects per 100 nm^2, with variations within the same surface preparation. Further, the concentration of type (i) defects remained in the same range when extending the annealing time up to 40 min at 650 °C or after annealing in O_2 ($5 \cdot 10^{-8}$ mbar, 10 min) at the same temperature. In Figure S1(d) an STM image taken after O_2 annealing is shown. Note that after annealing in O_2 no increase of type (ii) defects is expected as the O_2 was turned off when the sample was still at approximately 300 °C, while type (ii) defects accumulate on the surface at room temperature.

To minimize the risk of contaminating the surface by re-adsorption from the background pressure the annealing time and temperature was kept as low as possible to achieve a well-ordered surface: 10 min at 650 °C.

For all the annealing temperatures used here the LEED pattern is of similar quality.

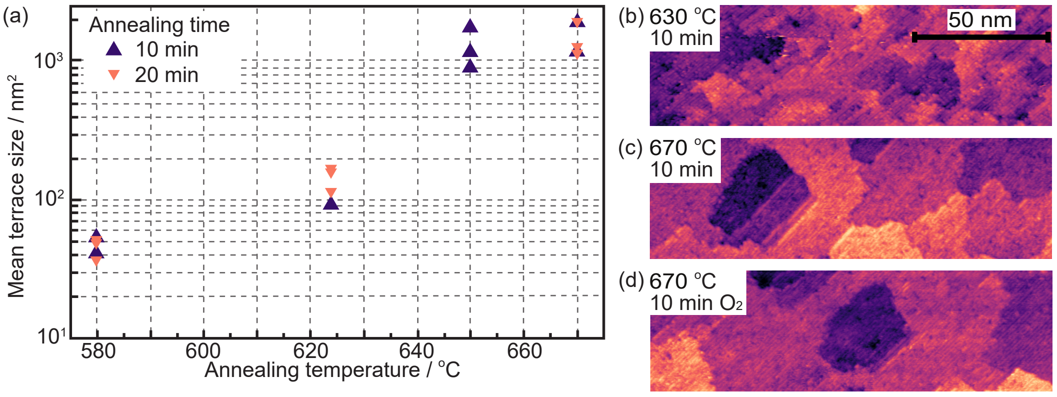

Figure S1. (a) Average terrace size as a function of annealing temperature and time, as observed in STM. (b) - (d) STM images of different surface preparations. (b) Annealing for 10 min at 630 °C. I=0.2 nA, U=2.1 V. (c) Annealing for 10 min at 670 °C. I=0.2 nA, U=1.6 V. (d) Annealing in $1 \cdot 10^{-7}$ mbar of O_2 for 10 min at 670 °C. I=0.2 nA, U=1.6 V. All STM images are 150×45 nm^2.

OH accumulation in vacuum

The O 1s spectra corresponding to the inset in Figure 2 are shown in Figure S2. The first spectra (in both normal and grazing emission) were measured directly after the surface preparation. The sample was then left in the measurement position and further spectra were measured at the indicated times. The pressure in the chamber was $1 \cdot 10^{-10}$ mbar. For reference the spectra after 1 L of D_2O exposure (i.e. saturation) are also shown. The two as prepared spectra and the spectrum after exposure to 1 L of D_2O are the same as in Figure 2. All spectra were fitted with the exact same components as those shown for the spectra in Figure 2, only the peak intensities were allowed to vary. The relative area of the OH component was calculated with the respective saturated spectra as reference.

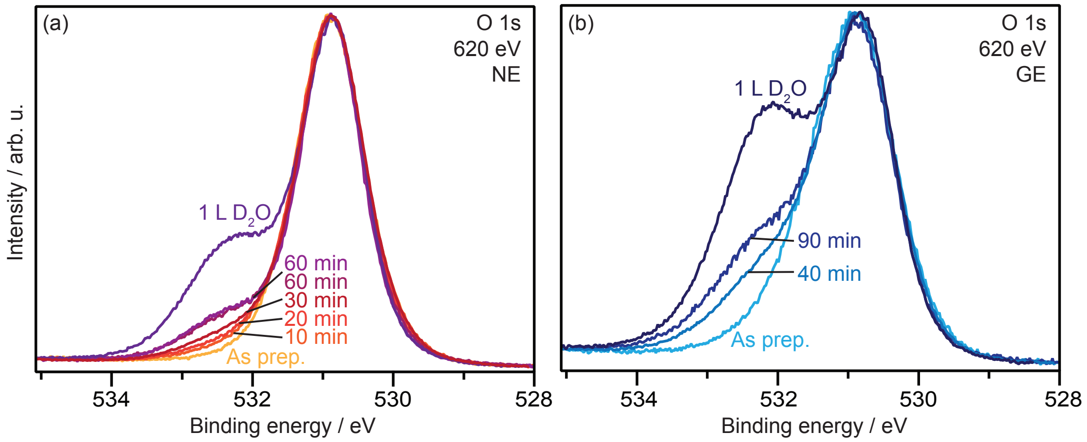

Figure S2. O 1s core level spectra of the ZnO(10$\bar{1}$4) surface measured after resting in $1 \cdot 10^{-10}$ mbar for the indicated times.

C 1s spectra of the as prepared and water exposed surface

The C 1s spectra corresponding to the O 1s spectra in Fig. 2 are shown in Fig. S3. On the as prepared surface in normal emission (NE) trace amounts of carbon are observed. In grazing emission (GE), however, no carbon is observed. That carbon is only visible in the measurements with deeper probing depth (i.e. NE) indicates that the small amount of carbon that is observed is not located at the surface.

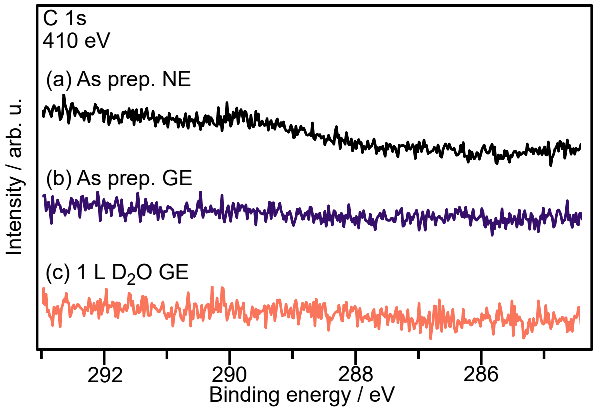

Figure S3. C 1s core level spectra of the ZnOZnO(10$\bar{1}$4). (a) As prepared, NE. (b) As prepared, GE. (c) After exposure to 1 L D_2O at room temperature, GE. The corresponding O 1s spectra are shown in Figure 2.

After exposure to 1 L D_2O at room temperature there is still no carbon present on the surface and we can exclude that the O_C component correspond to CO or CO_2 contaminants from residual gases. Further, in O 1s the components for CO and CO_2 is expected at even higher binding energies (535-540 eV)[1–3] and both CO and CO_2 mostly desorb below room temperature[4,5], though CO_2 has been shown to remain up to 320 K on steps in ultrathin ZnO[6].

Surface metallization: O 1s spectra in NE

The O 1s spectra of the as prepared and D_2O exposed surface measured in normal emission are shown in Figure S4. After exposure the O_A and O_B peaks are seen to shift 0.17 eV. This is to be compared with the shift of 0.4 eV seen in grazing emission.

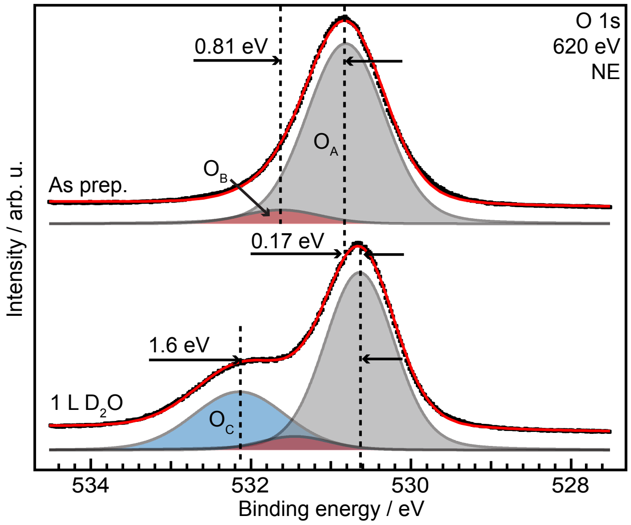

Figure S4. O 1s spectra of the as prepared surface (upper) and after exposure to 1 L D_2O at room temperature (lower) measured in NE. Black dots: measured data. Red line: fitted spectra.

SXRD models and fits of the bare surface

In Figure S5 the three models used as starting points for the structural refinement in the SXRD fits are shown. A systematic error of 15 % was assumed during the fitting. The orthorhombic unit cell determined from the SXRD measurements has the cell parameters: a = 12.40 Å, b = 3.29 Å, c = 40.32 Å. The ac-plane contains a mirror plane and the data were merged using space group Pm, which resulted in an agreement of 0.14. The fitting was performed by minimizing the χ^2, i.e. the difference between the calculated and measured structure factors through optimization of the displacement in the x- and z-directions for the Zn- and O- atoms in the first seven layers. Atoms in the deeper layers were fixed to their bulk positions. χ^2 is given by

$$\chi^2 = \frac{1}{N-p-1}\sum_i \frac{(|F_{obs}|-|F_{calc}|)^2}{\sigma^2(|F_{obs}|)}$$

where N is the number of points, p the number of free parameters, σ the error, and F_{obs} and F_{calc} are the measured and calculated structure factors, respectively. The best fits were obtained by accounting for atomic scale roughness. The used unit cell and crystallographic analysis does not implicitly include roughness on a larger lateral length scale, such as that arising due to a step and terraces structure. By including an explicit roughness term in the CTR calculation[7], one can account for this effect. The best fit results were obtained with a fitted r.m.s. roughness of 1.4 Å, in very good agreement with the STM measurements.

The fits corresponding to the lowest χ^2 in Table 1 in the article are shown in Figure S6, and the fitted displacements of the best fit, i.e. Model A, are shown in Table S1. The xyz-files for the three models are available in Supplementary Data 1 (Model A), 2 (Model B) and 3 (Model C). During the fitting the Debye-Waller factors of all atoms were kept fixed to their bulk values of 0.651 Å^2 and 0.64 Å^2 for the Zn- resp. O-atoms[8,9]. Fitting or manually modifying the factors had no significant influence on the χ^2 values. Starting from the optimized Model A, also the occupancy of the Zn-O pairs, as indicated in Figure 3(a) in the main article, corresponding to Model B and C were fitted. The optimal occupancy were found to be 0.98 and 1 respectively, where 1 in both cases corresponds to the pure Model A. This only gave minor improvements in the χ^2, from 1.65 for the pure Model A to 1.63 with the occupancy fitted.

Table S1. Displacements of the atoms in the best fit of the SXRD data [Model A]. The numbers of the atoms correspond to those shown in Figure S5(a).

Atom	Rel x-disp	Rel z-disp	Atom	Rel x-disp	Rel z-disp
Zn1	-0.0129	-0.0001	O1	-0.0152	-0.0027
Zn2	0.0052	-0.0028	O2	0	0
Zn3	0.0191	-0.0046	O3	0.0077	-0.0036
Zn4	0.0152	-0.0009	O4	0.0131	-0.005
Zn5	-0.0023	-0.0003	O5	0	0
Zn6	-0.0013	0.0019	O6	0.0122	0.0033
Zn7	0.0029	0.0007	O7	0	0
Zn8	0.0004	-0.0043	O8	-0.0006	-0.0016
Zn9	0.005	-0.0006	O9	0	0
Zn10	0.0029	-0.0003	O10	-0.002	0.0006
Zn11	0.0021	-0.0007	O11	-0.0142	-0.0046
Zn12	0.0019	0.0017	O12	0.0077	0.0015
Zn13	-0.0003	-0.0009	O13	0.0029	0.001
Zn14	0	-0.0011	O14	0.0041	0
Zn15	0.0033	0.0004	O15	0	-0.0002

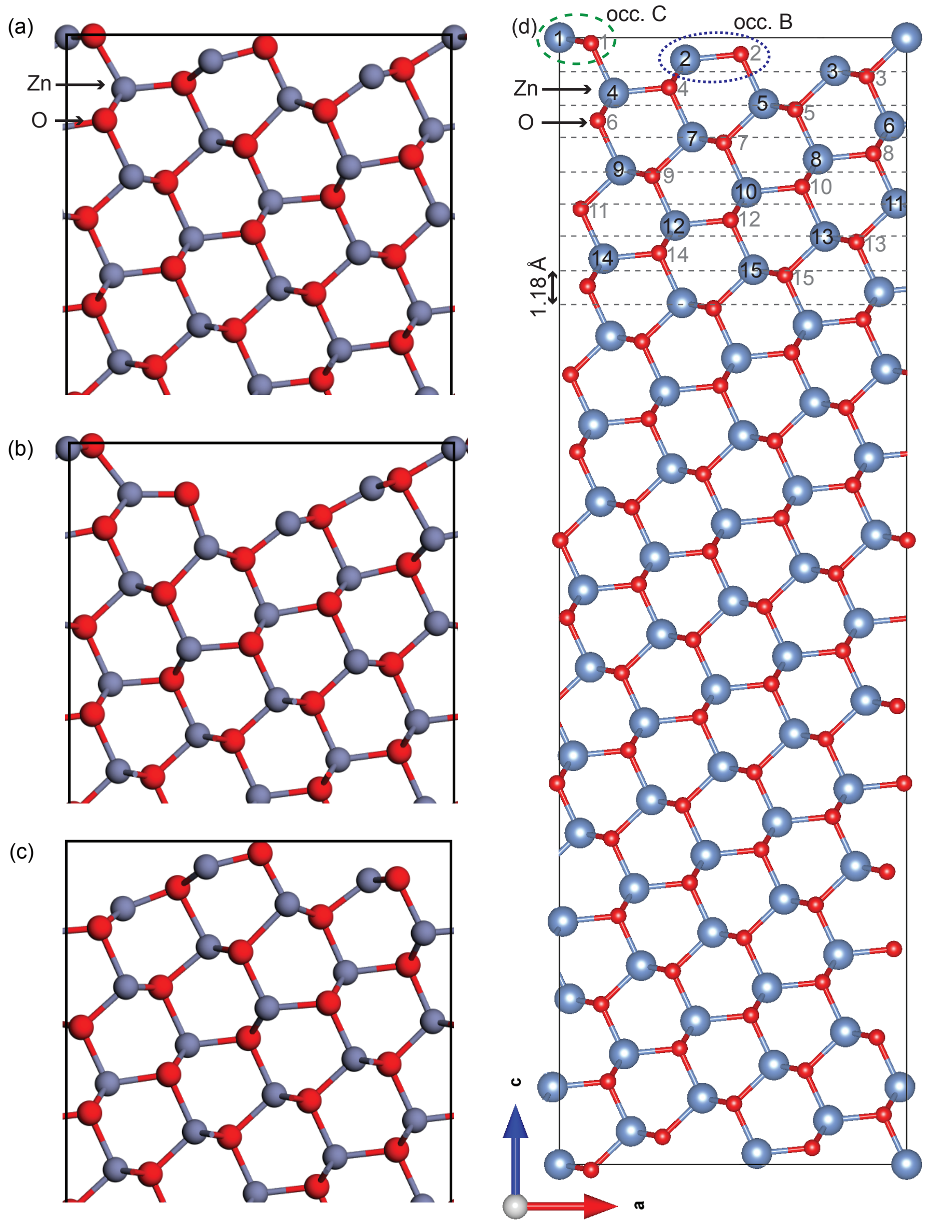

Figure S5. The first few layers of the three models A, B, and C with DFT relaxations. (d) The full unit cell of Model A used for the SXRD fitting (unrelaxed). The numbers in (d) correspond to the numbers of the atoms in Table S1. The dashed lines indicate the layer spacing in ZnO(10$\bar{1}$4).

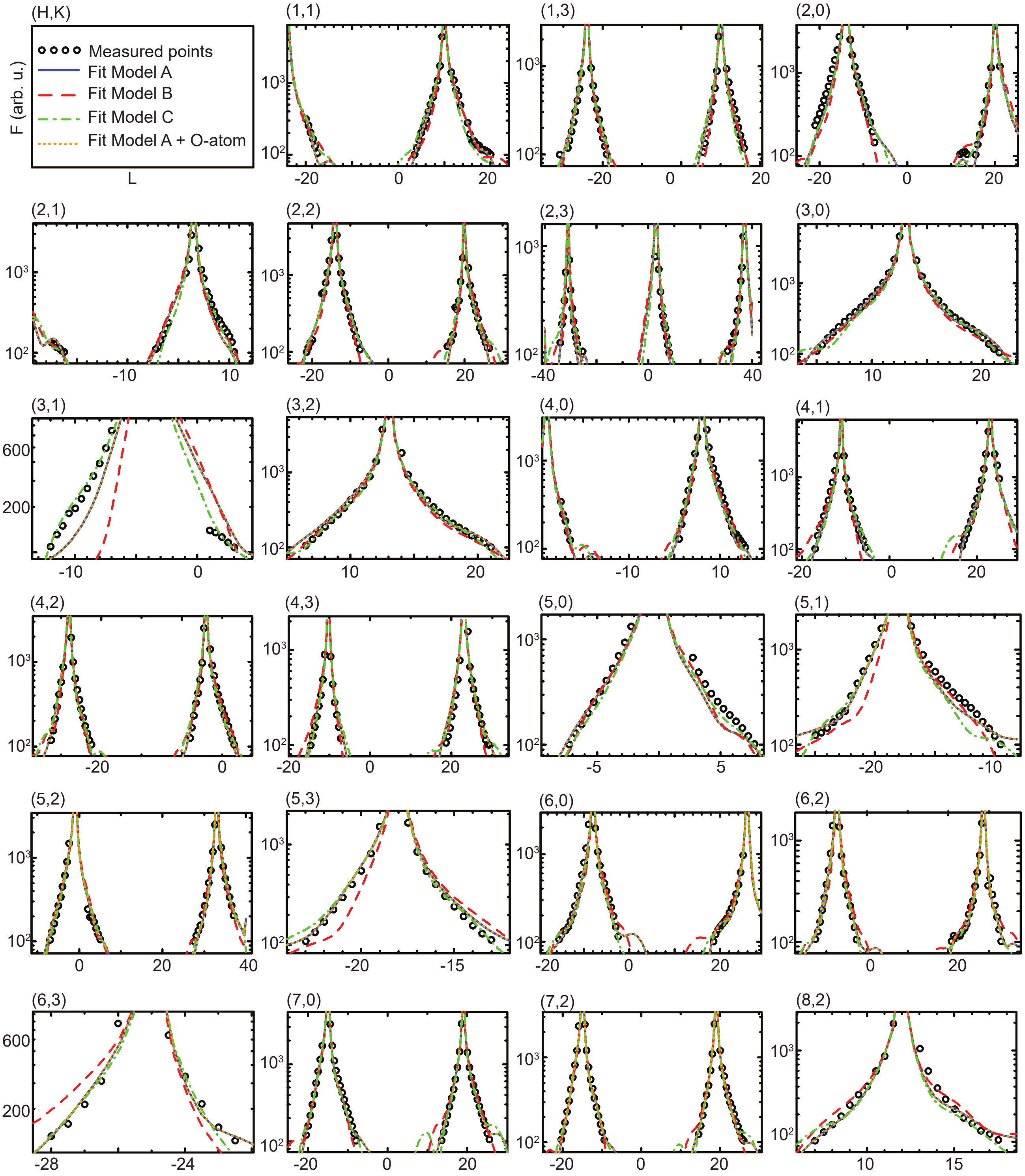

Figure S6. Best fits for the three models. χ^2_{norm} for model A, B and C are 1.65, 2.95, and 2.14 respectively.

DFT calculated core level shifts for Models B and C

The core level shifts of Models B and C are reported in Figure S7.

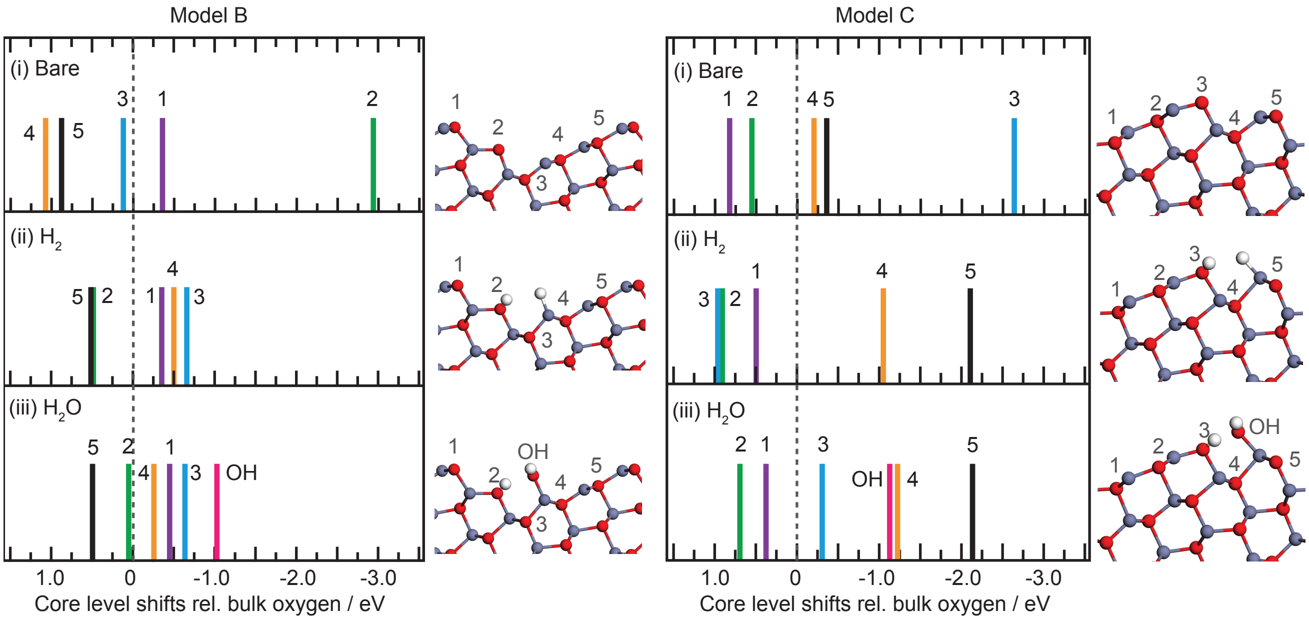

Figure S7. Surface O 1s core level shifts with respect to a bulk atom for models B (left) and C (right). The panels show the shifts for the case of the bare (i) surface together with the cases of one adsorbed H_2 (ii) and one adsorbed H_2O (iii). The numbering of the shifts follow the numbering in the structural models. Atomic color code: H (white), O (red), Zn (blue).

Comparison to O 1s in MgO(100)

Considering that oxygen both in bulk ZnO and MgO are in the same formal oxidation state of -II and that the O 1s binding energies in bulk ZnO and MgO are comparable, the XPS results indicate significant differences in the chemical bonding in both oxides. Oxygen in MgO is coordinated to 8 Mg^{2+} ions in a cubic geometry, which is a typical sign of a purely ionic bonding situation. Further support for the purely ionic nature of the interactions between Mg^{2+} and O^{2-} in MgO come from the complete transfer of two electrons from Mg to O in MgO as given by a Bader analysis and the analysis of the Density overlap region indicator (DORI)[10,11]. In ZnO, oxygen is coordinated by only four Zn ions in a tetrahedral geometry which is the typical coordination geometry of a sp^3 hybrid. Additionally, Bader charges indicate only a partial charge transfer of 1.25 electrons from Zn to O and the analysis of the corresponding DORI indicates the presence of a partially covalent bond. A similar degree of covalency is present in the Zn-OH bond. These significant differences in the nature of the chemical bonding between the two oxides can be expected to result in a different CLS of surface bonded OH-groups. Furthermore, the surface oxygens are, in contrast to a purely ionic compound such as MgO, not isolated but instead coupled through trans-ligand effects[11].

The DORI analysis is shown in Figure S8. The DORI-map ranges from zero (red) to one (blue). A value of one corresponds to charge accumulation owing to overlap of atomic densities. The bonding in the bulk of ZnO and MgO is found to be of different character. ZnO shows a bond-signature similar to covalent bonds[10], whereas MgO is of ionic character. Interestingly, the same pattern applied for the bonding to OH groups. Figures S8(c) and S8(d) shows the full H_2O coverage case for ZnO($10\bar{1}4$) and MgO(100). OH-groups are present in both cases originating from dissociated water. The character of the bond to the cation have a covalent feature for ZnO, whereas it is of ionic character on MgO.

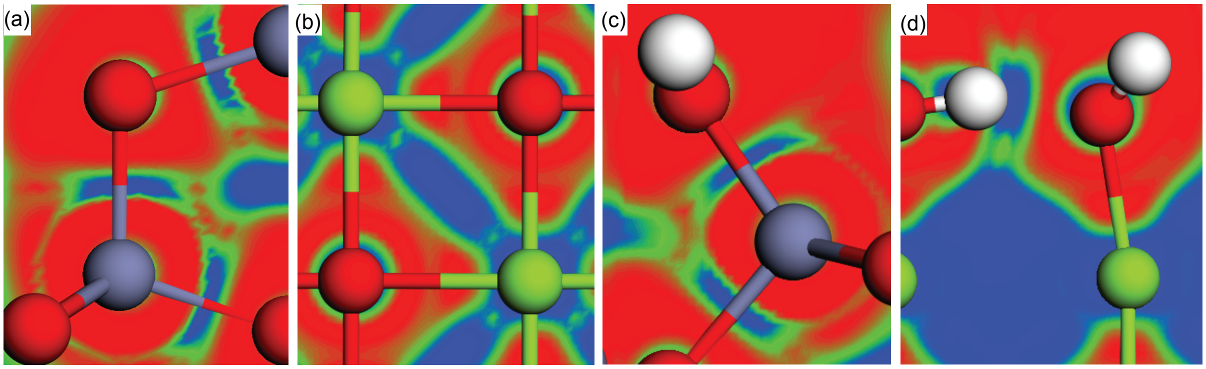

Figure S8. DORI analysis of bulk ZnO (a), bulk MgO (b), OH-group on ZnO(10$\bar{1}$4) (c) and OH-group on MgO(100).

SXRD fit with additional O-atom

As the DFT calculations suggest the presence of dissociated water the CTR data was fitted with an additional O-atom in the position of the OH group suggested by DFT. For Model A this lead to some adjustments of the atomic displacements in particular in the first layers (Table S2) and an improvement of the χ^2 to 1.52 (1.65 without the additional O-atom). The fit is displayed in Figure S6, the xyz-file is provided in Supplementary Data 4 and a table of the calculated and experimental structure factors are provided in Supplementary Data 5.For models B and C the addition of an O-atom lead to a worse χ^2.

Though DFT and SXRD agree on that the best fitting model is the bulk truncated structure with dissociated water adsorbed on the surface some discrepancies between the displacements (max 0.2 Å) in the fitted and calculated models remain. We tentatively attribute this to the restricted size of the DFT unit cell and the fact that the DFT calculations are performed at T=0 K. Systematic errors in the SXRD data analysis also contribute.

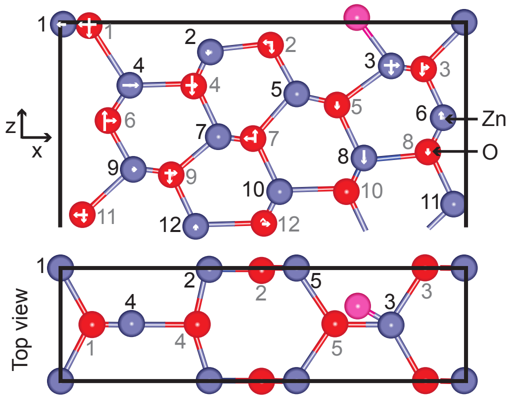

Figure S9. Model A with an additional O-atom corresponding to adsorbed OH. Color code: Blue - Zn, Red - O in ZnO. The numbers refer to Table S2.

DFT calculated displacements of atoms for the best fitting model

The calculated displacements with respect to the atoms' bulk positions for the DFT model with the lowest surface energy, i.e. Model A with dissociated water as displayed in Fig. 3 in article, are given in table S3.

Table S2. Displacements of the atoms of model (A) with one additional O atom.

Atom	Rel x-disp	Rel z-disp	Atom	Rel x-disp	Rel z-disp
Zn1	-0.0158	-0.001	O1	-0.0152	-0.0027
Zn2	0.0019	-0.0026	O2	0	0
Zn3	0.0205	-0.0043	O3	0.0077	-0.0036
Zn4	0.0182	-0.0003	O4	0.0131	-0.005
Zn5	-0.0029	-0.0003	O5	0	0
Zn6	-0.0015	0.0016	O6	0.0122	0.0033
Zn7	0.0029	0.0007	O7	0	0
Zn8	0.0004	-0.0043	O8	-0.0006	-0.0016
Zn9	0.005	-0.0006	O9	0	0
Zn10	0.0029	-0.0003	O10	-0.002	0.0006
Zn11	0.0021	-0.0007	O11	-0.0142	-0.0046
Zn12	0.0019	0.0017	O12	0.0077	0.0015
Zn13	-0.0003	-0.0009	O13	0.0029	0.001
Zn14	0	-0.0011	O14	0.0041	0
Zn15	0.0033	0.0004	O15	0	-0.0002

Table S3. Calculated atomic displacements in Å for Model A with dissociated water.

Atom	x	y	z	Atom	x	y	z	Atom	x	y	z	Atom	x	y	z
Zn1	-0.01	0.09	-0.15	O1	-0.21	0.09	0.09	Zn34	-0.05	0.01	0.04	O34	-0.05	0.02	0.03
Zn2	0.1	-0.07	-0.18	O2	-0.09	0.11	0.04	Zn35	-0.05	0.01	0.04	O35	-0.05	0.01	0.03
Zn3	-0.19	0.14	0.23	O3	-0.17	0.16	0.12	Zn36	-0.05	0.01	0.04	O36	-0.04	0.01	0.03
Zn4	-0.13	0.04	0.18	O4	-0.1	-0.05	0.13	Zn37	-0.05	0.01	0.03	O37	-0.05	0.01	0.03
Zn5	-0.01	0.1	0.1	O5	-0.07	0.09	0.03	Zn38	-0.05	0.01	0.03	O38	-0.04	0.01	0.03
Zn6	-0.14	0.09	0.13	O6	-0.1	0.04	0.12	Zn39	-0.04	0.01	0.03	O39	-0.04	0.01	0.03
Zn7	-0.1	0.01	0.03	O7	-0.11	0.02	0.12	Zn40	-0.04	0.01	0.03	O40	-0.04	0.01	0.03
Zn8	-0.07	0.06	0.08	O8	-0.07	0.08	0.08	Zn41	-0.04	0.01	0.03	O41	-0.04	0.01	0.03
Zn9	-0.14	0.04	0.12	O9	-0.11	0.03	0.09	Zn42	-0.04	0.01	0.03	O42	-0.04	0.01	0.02
Zn10	-0.08	0.03	0.1	O10	-0.08	0.06	0.06	Zn43	-0.04	0.01	0.03	O43	-0.03	0.01	0.02
Zn11	-0.09	0.05	0.08	O11	-0.08	0.05	0.07	Zn44	-0.03	0.01	0.03	O44	-0.03	0.01	0.02
Zn12	-0.1	0.03	0.09	O12	-0.1	0.03	0.1	Zn45	-0.04	0.01	0.02	O45	-0.03	0.01	0.02
Zn13	-0.08	0.04	0.07	O13	-0.07	0.03	0.06	Zn46	-0.03	0.01	0.02	O46	-0.03	0.01	0.02
Zn14	-0.09	0.03	0.07	O14	-0.09	0.03	0.07	Zn47	-0.03	0.01	0.02	O47	-0.03	0.01	0.02
Zn15	-0.1	0.02	0.09	O15	-0.09	0.03	0.07	Zn48	-0.03	0.01	0.02	O48	-0.03	0.01	0.02
Zn16	-0.08	0.03	0.07	O16	-0.07	0.03	0.06	Zn49	-0.03	0.01	0.02	O49	-0.02	0.01	0.02
Zn17	-0.09	0.03	0.07	O17	-0.08	0.02	0.07	Zn50	-0.03	0.01	0.02	O50	-0.03	0.01	0.02
Zn18	-0.08	0.03	0.07	O18	-0.08	0.03	0.05	Zn51	-0.02	0.01	0.02	O51	-0.02	0	0.01
Zn19	-0.08	0.02	0.06	O19	-0.08	0.02	0.06	Zn52	-0.02	0.01	0.02	O52	-0.02	0.01	0.01
Zn20	-0.07	0.02	0.07	O20	-0.07	0.02	0.05	Zn53	-0.02	0.01	0.02	O53	-0.02	0.01	0.01
Zn21	-0.07	0.02	0.05	O21	-0.07	0.02	0.05	Zn54	-0.02	0	0.02	O54	-0.02	0.01	0.01
Zn22	-0.07	0.02	0.06	O22	-0.07	0.02	0.05	Zn55	-0.02	0.01	0.01	O55	-0.02	0	0.01
Zn23	-0.07	0.02	0.05	O23	-0.07	0.01	0.05	Zn56	-0.01	0	0.01	O56	-0.01	0.01	0.01
Zn24	-0.07	0.02	0.05	O24	-0.07	0.02	0.05	Zn57	-0.02	0.01	0.01	O57	-0.01	0	0.01
Zn25	-0.07	0.02	0.05	O25	-0.06	0.01	0.05	Zn58	-0.02	0	0.01	O58	-0.01	0.01	0.01
Zn26	-0.07	0.01	0.05	O26	-0.06	0.02	0.04	Zn59	-0.01	0	0.01	O59	-0.01	0	0
Zn27	-0.06	0.02	0.05	O27	-0.06	0.01	0.04	Zn60	-0.01	0	0.01	O60	-0.01	0	0
Zn28	-0.06	0.01	0.05	O28	-0.06	0.02	0.04	Zn61	-0.01	0.01	0.01	O61	0	0	0
Zn29	-0.06	0.02	0.05	O29	-0.06	0.01	0.04	Zn62	-0.01	0	0.01	O62	-0.01	0.01	0
Zn30	-0.06	0.01	0.04	O30	-0.06	0.02	0.04	Zn63	-0.01	0	0	O63	0	0	0
Zn31	-0.06	0.01	0.04	O31	-0.05	0.01	0.04	Zn64	0	0	0	O64	0	0	0
Zn32	-0.05	0.01	0.04	O32	-0.05	0.01	0.04	Zn65	0	0	0	O65	0	0	0
Zn33	-0.05	0.02	0.04	O33	-0.05	0.01	0.03	Zn66	0	0	0	O66	0	0	0

Additional H_2O on Model A

The adsorption of additional H_2O molecules onto Model A was investigated by considering a coverage of two and three molecules in the (1×1) surface cell. As seen in the phase diagram (Figure 3) the case with three molecules is favoured. This coverage corresponds to one molecule per under-coordinated Zn-ion. Because of the structural complexity with the possibility of several close to degenerate structures, 43 initial structures were geometrically optimized. The initial structures included situations with one, two and three intact H_2O molecules. The relaxed structures had all either one or two dissociated H_2O molecules. A range of different structures were obtained within 0.1 eV. Four examples are given in Figure S10 (a) and (b) have two intact H_2O molecules, whereas (c) and (d) have one intact molecule.

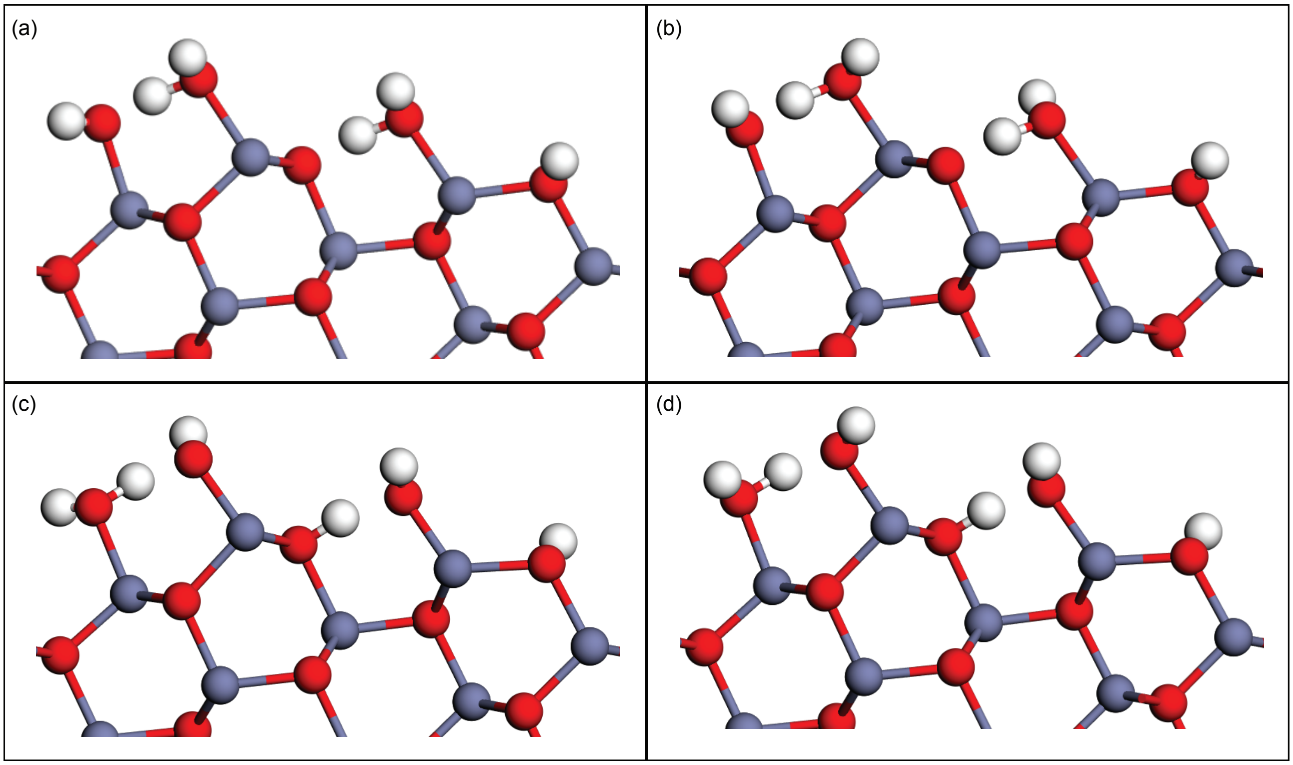

Figure S10. Examples of surface structures for three H_2O adsorbed in the (1×1) surface cell. The different structures are close in energy (within 0.05 eV). (a) and (b) have two intact H_2O molecules, whereas (c) and (d) have one intact molecule. Atomic color code: H (white), O (red), Zn (blue).

XPS overview spectrum

In Figure S11 an overview spectrum taken in normal emission on the as prepared $ZnO(10\bar{1}4)$ crystal is shown. No contaminants are observed.

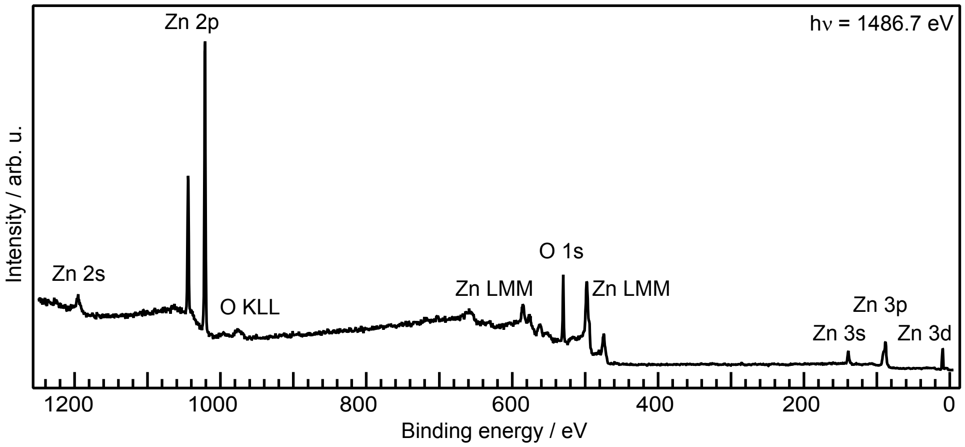

Figure S11. Overview spectrum from the as prepared $ZnO(10\bar{1}4)$ crystal.

Supplementary References

1. Akhter, S., Lui, K. & Kung, H. H. Comparison of the chemical propertles of the zinc-polar, the oxygen-polar, and the nonpolar surfaces of ZnO. J. Phys. Chem. **89**, 1958–1964, DOI: 10.1021/j100256a029 (1985).
2. Au, C. T., Hirsch, W. & Hirschwald, W. Adsorption of carbon monoxide and carbon dioxide on annealed and defect zinc oxide (000-1) surfaces studied by photoelectron spectroscopy (xps and ups). Surf. Sci. **197**, 391–401 (1988).
3. Becker, T., Kunat, M., Boas, C., Burghaus, U. & Wöll, C. Adsorption dynamics of co on the polar surfaces of zno. J. Chem. Phys. **113**, 6334, DOI: 10.1063/1.1309131 (2000).
4. Wang, J., Hokkanen, B. & Burghaus, U. Adsorption of co2 on pristine zn–zno(0001) and defected zn–zno(0001): A thermal desorption spectroscopy study. Surf. Sci. **577**, 158–166, DOI: 10.1016/j.susc.2004.12.030 (2005).
5. Wang, Y. et al. Co2 activation by zno through the formation of an unusual tridentate surface carbonate. Angewandte Chemie Int. Ed. DOI: 10.1002/anie.200700564 (2007).
6. Deng, X., Sorescu, D. C. & Lee, J. Enhanced adsorption of CO2 at steps of ultrathin ZnO: the importance of zn–o geometry and coordination. Phys. Chem. Chem. Phys. **19**, 5296–5303, DOI: 10.1039/c6cp08379j (2017).
7. Vlieg, E. ROD: a program for surface x-ray crystallography. J. Appl. Crystallogr. **33**, 401–405, DOI: 10.1107/s0021889899013655 (2000).
8. Sawada, H., Wang, R. & Sleight, A. W. An electron density residual study of zinc oxide. J. Solid State Chem. **122**, 148–150, DOI: 10.1006/jssc.1996.0095 (1996).
9. Schlepütz, C. M. et al. The presence of a (1x1) oxygen overlayer on ZnO(0001) surfaces and at schottky interfaces. J. Physics: Condens. Matter **24**, 095007, DOI: 10.1088/0953-8984/24/9/095007 (2012).
10. de Silva, P. & Corminboeuf, C. Simultaneous visualization of covalent and noncovalent interactions using regions of density overlap. J. Chem. Theory Comput. **10**, 3745, DOI: 10.1021/ct500490b (2014).
11. Busch, M., Wang, R. B., Hellman, A., Rossmeisl, J. & Grönbeck, H. The influence of inert ions on the reactivity of manganese oxides. J. Phys. Chem. C **122**, 126, DOI: 10.1021/acs.jpcc.7b10760 (2018).
