# Supplementary material for: Role of hydroxylation for the atomic structure of a non-polar vicinal zinc oxide
Source: Commun Chem. 2021 Jan 20;4:7. doi: 10.1038/s42004-020-00442-6 (PMC9814410; doi:10.1038/s42004-020-00442-6)
Supplement: Supplementary file 2 — Description of Additional Supplementary Files [file 42004_2020_442_MOESM2_ESM.pdf]

Description of Additional Supplementary Files

File name: Supplementary Data 1
Description: Structure of the bare Model A, used for the SXRD fits.

File name: Supplementary Data 2
Description: Structure of the bare Model B, used for the SXRD fits.

File name: Supplementary Data 3
Description: Structure of the bare Model C, used for the SXRD fits.

File name: Supplementary Data 4
Description: Structure of Model A with one additional O-atom, used for the SXRD fits.

File name: Supplementary Data 5
Description: Comparison of the measured and calculated structure factors for the full set of CTRs.
